# Poster Abstract: Protecting User Data Privacy with Adversarial Perturbations

**DOI:** 10.1145/3412382.3458776

**Published:** 2021-05

**Authors:** Ziqi Wang, Brian Wang, Mani Srivastava

**Affiliations:** University of California, Los Angeles, Los Angeles, USA

## Abstract

The increased availability of on-body sensors gives researchers access to rich time-series data, many of which are related to human health conditions. Sharing such data can allow cross-institutional collaborations that create advanced data-driven models to make inferences on human well-being. However, such data are usually considered privacy-sensitive, and publicly sharing this data may incur significant privacy concerns. In this work, we seek to protect clinical time-series data against membership inference attacks, while maximally retaining the data utility. We achieve this by adding an imperceptible noise to the raw data. Known as adversarial perturbations, the noise is specially trained to force a deep learning model to make inference mistakes (in our case, mispredicting user identities). Our preliminary results show that our solution can better protect the data from membership inference attacks than the baselines, while succeeding in all the designed data quality checks.

## 1 INTRODUCTION

Clinical time series datasets collected from on-body sensors, combined with machine learning, can enable powerful health-related inferences about individuals or large populations. These models are often data-hungry, so making clinical datasets publicly available can help the training of such models. However, accesses to these clinical datasets are often restricted as they contain sensitive information about their contributors regarding health conditions.

To address the privacy concerns, one potential approach is synthetic generation: authentic raw data is used to generate a synthetic dataset that obfuscates the identities of the users and can be shared publicly. Meanwhile, the utility of the time series data should still be maintained so that the data remains useful for making health inferences. In 2020, the NeurIPS Hide-and-Seek Privacy Challenge [2] is proposed to develop synthetic generation algorithms. In the challenge, teams are organized into two tracks: hiders and seekers.

Hiders generate synthetic data from the AmsterdamUMCdb dataset, which contains data entries (multimodal time series) from ~ 20000 ICU patients. In this work, we propose a hider algorithm that uses adversarial perturbations to protect user privacy while retaining utility. It is inspired by previous works on adversarial attacks [1], where the performance of a neural model drops significantly because of an imperceptible noise added to the raw data. In our case, the adversarial perturbations are trained against a user recognition network to hide the identity-related information.

This competition [2] formulates privacy as robustness to membership inference attacks, where the goal of the seeker (attacker) is to infer whether or not a given data entry is used to generate the synthetic dataset. User identities in an ideal synthetic dataset should be independent from that of the raw dataset, i.e. the synthesizing process should create new users that never exist. In the challenge, a subset of users in the raw dataset is used for synthetic generation. If they can be successfully identified by a membership inference attack, then there is an association between identities of these users and the synthetic users, indicating a privacy risk.

**Figure 1:**
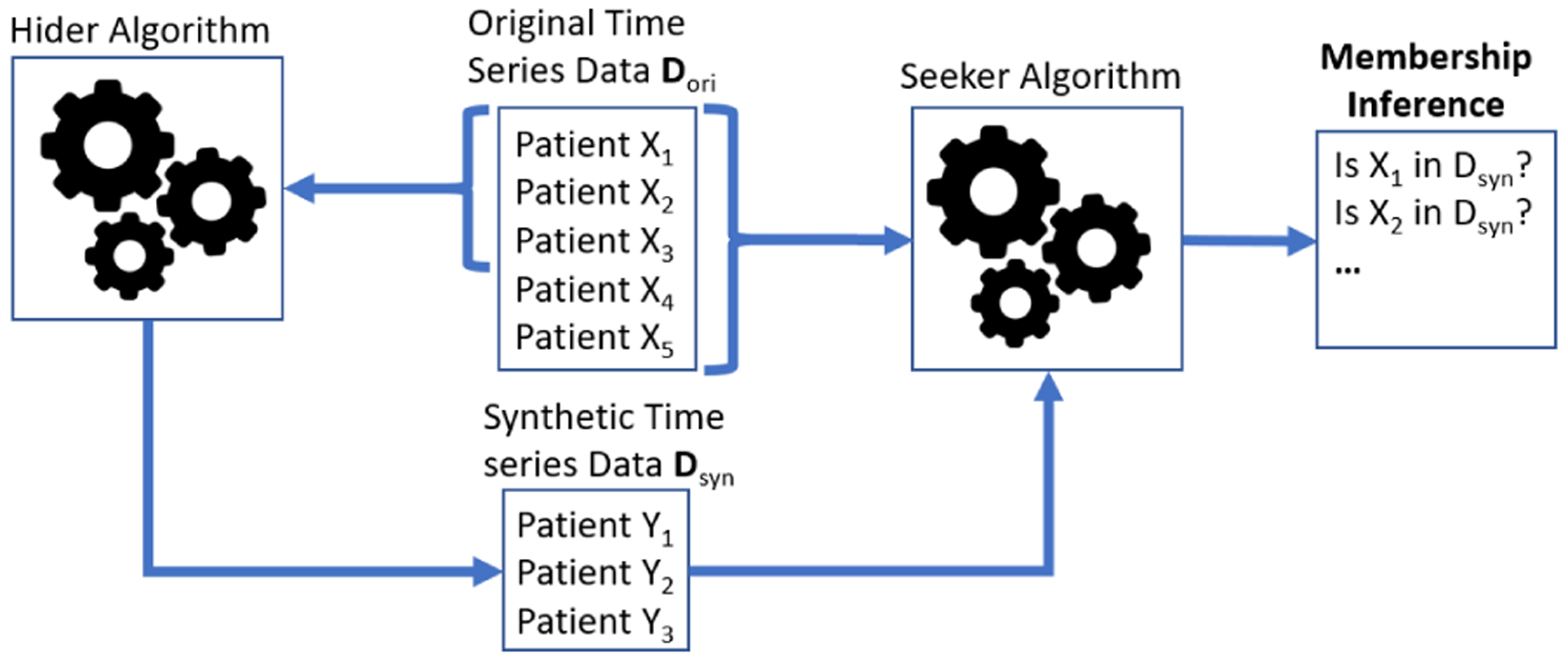
Example membership inference attacks in the context of this competition.

We provide an example in Figure 1. Our approach resides on the hider side, where a synthetic dataset *D*_*syn*_ is generated from a subset of patients {*X*_1_*, X*_2_*, X*_3_} of the original dataset *D*_*ori*_. The seekers (attackers) have access to both the original dataset *D*_*ori*_ and the synthetic dataset *D*_*syn*_. A successful seeker (attacker) can identify that *X*_1_*, X*_2_*, X*_3_ is used in the synthetic generation, while the goal of our algorithm (hider) is to generate a *D*_*syn*_ that can prevent such attacks.

## 2 METHODS

Shan et. al [3] proposed an algorithm which made efforts to “poison” unauthorized facial recognition models by publishing photos on the social media with adversarial perturbations. These perturbations are trained to scramble the embeddings of different users in a neural network (NN) feature extractor. Inspired by this approach designed for images, we propose a synthetic time series data generation method to protect user data privacy by adding noise to the data, where the noise is trained in an adversarial manner.

**Figure 2:**
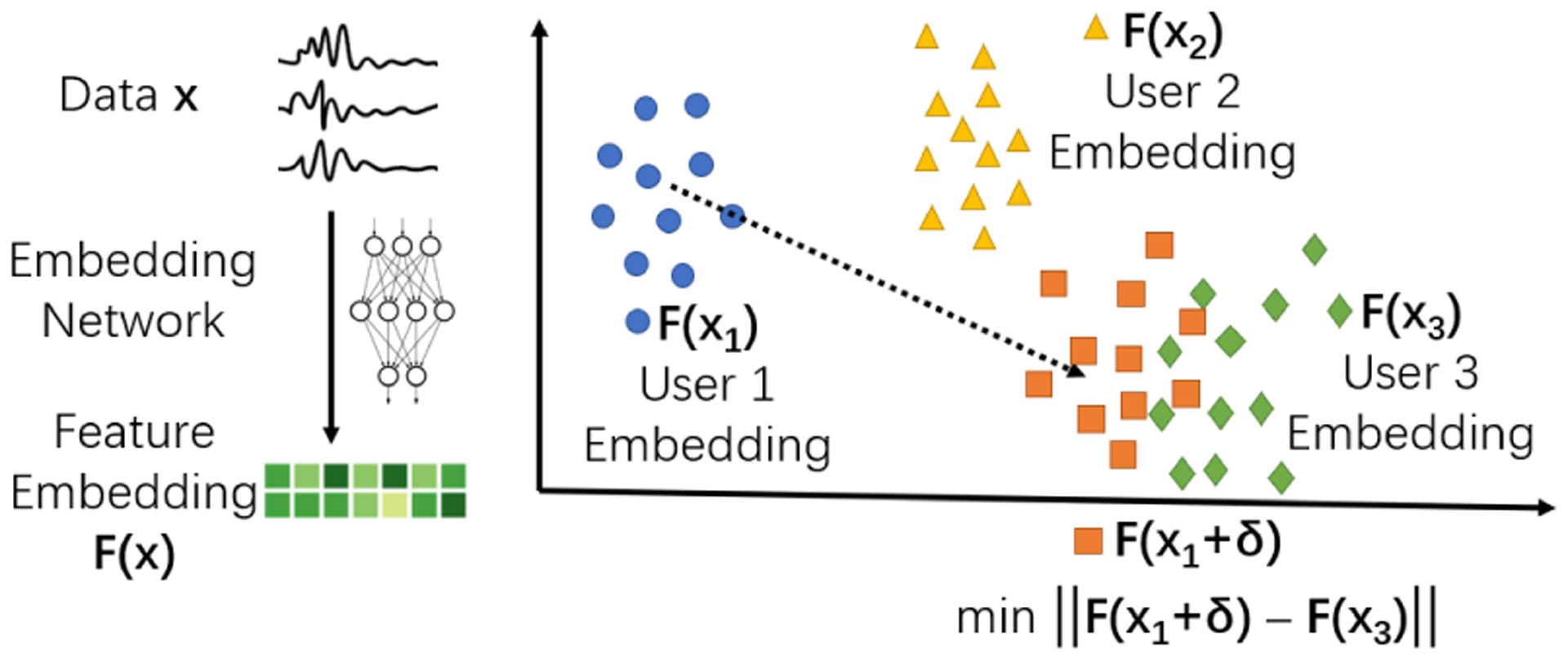
An overview of our proposed approach.

The high-level idea of our proposed method is visualized in Figure 2. A trained feature extraction neural network generates a feature embedding for each user’s time series. This embedding is trained to represent the user’s identity. A small perturbation in the raw time series may cause its embedding to change drastically because of the discontinuity of the function approximated by the neural network. The added noise is trained to “drag” the feature embedding of the current user towards the embedding of a different user, so that the network is confused about their identities. We have made our implementation publicly available.^1^

The algorithm can be described in three steps:

**Step 1:** Train a Convolutional Neural Network (CNN) feature extractor using a Siamese structure. The feature extractor works to recognize the user and maximize the embedding distances between different users using contrastive loss.
**Step 2:** Define a CNN with exactly the same structure as previously, except that at the input layer, we add a layer of the same dimension to inject additive noise. Then we copy and fix the trained CNN weights. The only learnable parameter is the added noise ***δ***.
**Step 3:** For each user (for example, data entry ***x***_**1**_ in Figure 2), select a data entry ***x***_**3**_ from another user, whose embedding ***F*** (***x***_**3**_) is distant from ***F*** (***x***_**1**_). Then we train the noise ***δ*** using gradient descent to move the current embedding ***F*** (***x***_**1**_ + ***δ***) towards embedding ***F*** (***x***_**3**_). Then ***x***_**1**_ +***δ***_***opt***_ is returned as the generated data entry. This process is repeated for all the users to scramble their embeddings.

## 3 EVALUATIONS

### 3.1 Baselines

We compare our synthetic generation algorithm against three baseline generators on the AmsterdamUMCdb dataset. The input data have timestamps and 70 features. After imputation. the data are chunked into segments of 100 time-steps to form matrices of 100×71. The first simple baseline, **Add Noise**, adds an i.i.d. Gaussian noise to every value in the dataset with zero mean and a standard deviation as a hyper-parameter. The second baseline, **TimeGAN** [4], seeks to use Generative Adversarial Networks to capture temporal dynamics of the time series and generate new data. The third baseline, **Genetic**, is inspired by [1]. This approach uses the genetic algorithm, mutating user data by adding noise. In this baseline we train several models checking the utility of mutated data for the natural selection process.

### 3.2 Metrics

Each data entry has two dimensions: features and time. Our work employs two utility metrics on each dimension to check the data quality. The first metric is **Feature Prediction Error**. A feature predictor is a NN trained to predict a missing feature from the rest of the features. The second metric is **One-step-ahead Prediction Error**. In this case, a NN is trained to predict the value of all features at a particular time step from the previous time steps.

In both metrics, we train two parallel models using the original dataset *D*_*ori*_ and the synthetic data *D*_*syn*_ separately. Some testing data are held back from *D*_*ori*_, on which we can check the performance of the two predictors. The prediction errors on the testing data are measured by root mean squared error (RMSE). The test RMSE of the model trained with *D*_*syn*_ should be less than a threshold of the RMSE given by the model trained with *D*_*ori*_, in order to pass the utility test. In this work, we pick the threshold to be 5x, in accordance with the competition guidelines.

In terms of privacy, we use the maximum re-identification (Re-ID) score across all nine seekers participating in this challenge as the metric. The Re-ID score is the percentage of the correct re-identifications made by a seeker.

### 3.3 Results

Our preliminary results are shown in Table 1. We evaluate the performance from two aspects: utility and privacy. In the feature prediction test, only our proposed adversarial perturbation method passed all tests compared to the baselines. In terms of the one-step-ahead prediction, all the four methods pass. The last column, Re-ID score, shows the performance of privacy protection where a lower score is better. Our proposed method outperforms all 3 baselines.

As a conclusion, we propose a synthetic data generation method to protect the user data privacy while retaining data quality. We perturb raw time series data to scramble their embeddings in a user identification neural network to hide identity-related information. The perturbation is regularized so that the data utility is minimally affected. This effort is one step towards cross-institutional health data sharing, which is expected to have a positive social impact.

## Figures and Tables

**Table 1:** Evaluation Results

Data Generator	Feature Prediction	One-step-ahead Prediction	Re-ID Score
**Add Noise**	8/10 Pass	Pass	0.5734
**TimeGAN**	6/10 Pass	Pass	0.5047
**Genetic**	8/10 Pass	Pass	0.6315
**Adversarial(Ours)**	**10/10 Pass**	Pass	**0.5037**

## Footnotes

1
https://github.com/nesl/hide-and-seek-challenge
